# Long-term potentiation and the role of *N*-methyl-d-aspartate receptors

**DOI:** 10.1016/j.brainres.2015.01.016

**Published:** 2015-09-24

**Authors:** Arturas Volianskis, Grace France, Morten S. Jensen, Zuner A. Bortolotto, David E. Jane, Graham L. Collingridge

**Affiliations:** a Center for Synaptic Plasticity, School of Physiology and Pharmacology, University of Bristol, United Kingdom; b Department of Biomedicine, University of Aarhus, Denmark

**Keywords:** *N*-methyl-d-aspartate, NMDA, *N*-methyl-d-aspartate receptors, NMDARs, Hippocampus, Long-term potentiation, LTP, Short-term potentiation, STP, Long-term depression, LTD

## Abstract

*N*-methyl-d-aspartate receptors (NMDARs) are known for their role in the induction of long-term potentiation (LTP). Here we start by reviewing the early evidence for their role in LTP at CA1 synapses in the hippocampus. We then discuss more recent evidence that NMDAR dependent synaptic plasticity at these synapses can be separated into mechanistically distinct components. An initial phase of the synaptic potentiation, which is generally termed short-term potentiation (STP), decays in an activity-dependent manner and comprises two components that differ in their kinetics and NMDAR subtype dependence. The faster component involves activation of GluN2A and GluN2B subunits whereas the slower component involves activation of GluN2B and GluN2D subunits. The stable phase of potentiation, commonly referred to as LTP, requires activation of primarily triheteromeric NMDARs containing both GluN2A and GluN2B subunits. In new work, we compare STP with a rebound potentiation (RP) that is induced by NMDA application and conclude that they are different phenomena. We also report that NMDAR dependent long-term depression (NMDAR-LTD) is sensitive to a glycine site NMDAR antagonist. We conclude that NMDARs are not synonymous for either LTP or memory. Whilst important for the induction of LTP at many synapses in the CNS, not all forms of LTP require the activation of NMDARs. Furthermore, NMDARs mediate the induction of other forms of synaptic plasticity and are important for synaptic transmission. It is, therefore, not possible to equate NMDARs with LTP though they are intimately linked.

*This article is part of a Special Issue entitled SI: Brain and Memory*.

## Highlights

• NMDARs confer important properties to the induction of LTP.
• STP comprises two components with different kinetics and NMDAR subtype dependence.
• GluN2A and GluN2B subunit containing NMDARs are responsible for the induction of LTP.
• NMDA-induced potentiation is distinct from STP and LTP.
• A specific glycine-site NMDAR antagonist blocks the induction of LTD.

## 1 Introduction

Long-term potentiation (LTP) has been extensively studied with the belief that the mechanisms involved in its generation are essentially the same as those that underlie the synaptic basis of memory (Bliss and Collingridge, 1993; Bliss et al., 2014). Thus by understanding LTP one might start to understand the molecular basis of learning and memory (Albright et al., 2000; Martin et al., 2000).

LTP, and its counterpart long-term depression (LTD), comprise a family of synaptic plastic processes. They are highly complex, befitting a set of mechanisms that provide the substrate for information storage in the mammalian brain. It is convenient to consider three components of LTP (and LTD): induction (the trigger mechanism); expression (what changes to result in an increase (or decrease) in synaptic transmission; and transduction (the signalling cascades that lead from induction to expression and maintain the alteration in synaptic efficiency). In the present article we focus on induction.

**Fig. 1:**
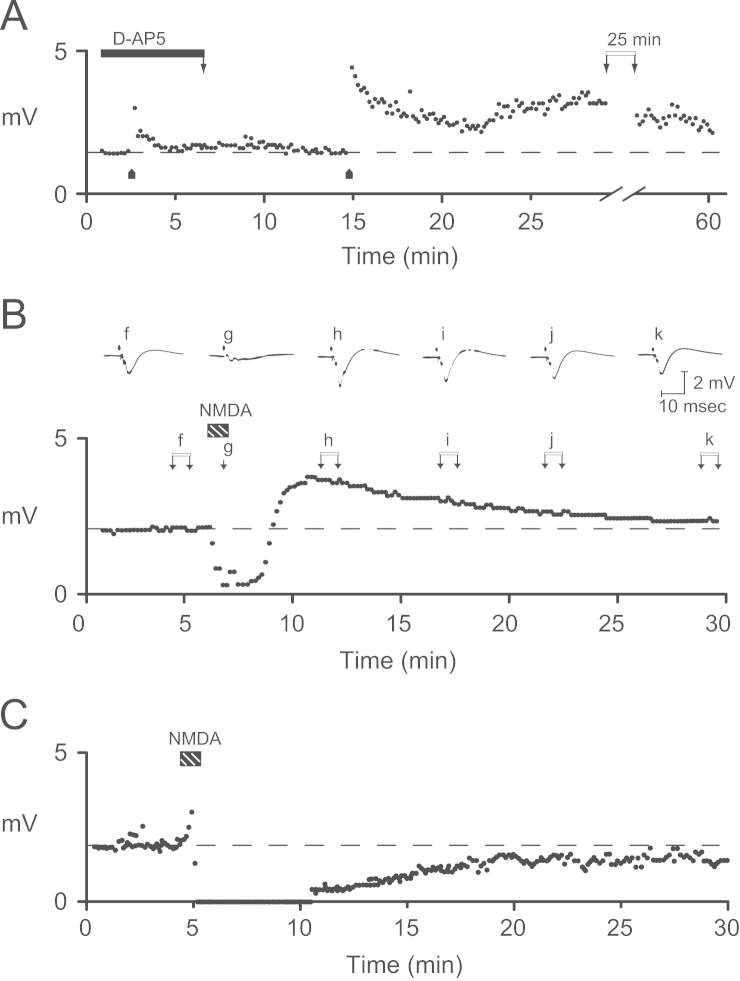
NMDAR-dependent synaptic plasticity. (A) The specific NMDAR antagonist AP5 (APV) blocks the induction of LTP. (B) Brief application of NMDA induces a transient depression of the FV and fEPSP followed by a RP of the fEPSP. NMDA was applied locally to the dendritic region of CA1 by passing a 50 nA current to a solution of *N-*methyl-dl-aspartate contained within an electrophoretic pipette. (C) Bath applied NMDA (50 µM, 1 min; applied as a racemate solution) induces LTD. Data replotted from Collingridge et al. (1983a, 1983b).

In the early 1980s we found that the *N*-methyl-d-aspartate receptor (NMDAR) is a trigger for the induction of LTP at the Schaffer collateral-commissural pathway (SCCP) in the hippocampus (Collingridge et al., 1983b) (Fig. 1A). Since then it has become clear that the NMDAR is the trigger for the induction of LTP at the majority, but not all, of the synapses that have so far been investigated in the CNS and that it is also the trigger for some forms of LTD too. It has also become apparent that the NMDAR is critically involved in many, but not all, forms of learning and memory (Bliss et al., 2014). As a consequence the NMDAR has become equated with LTP and learning and memory. This leads to the topic of this article in which we discuss various plasticity phenomena that are related to NMDA receptor activation, posing the question: Does NMDAR=LTP=Memory?

## 2 Properties of LTP

The phenomenon of LTP was first described in detail in two classic papers published in the Journal of Physiology in 1973 by Bliss and Lomo (1973) and Bliss and Gardner-Medwin (1973). These workers had been in search of the synaptic basis of learning and memory and were well aware that the phenomenon that they reported was a very attractive mnemonic device. They found that brief periods of high frequency stimulation (HFS), also commonly referred to as tetani, led to an enduring increase in the size of the synaptic potentials recorded in the dentate gyrus in response to stimulation of the perforant path projection from the entorhinal cortex. In anaesthetised rabbits the effect lasted for hours; in the freely moving animal for months. The persistence of LTP was one property that immediately fitted with the idea that LTP was engaging processes involved in long-term memory.

Their study, and the papers that followed soon afterwards, defined the hall-mark features of LTP: input-specificity, co-operativity and associativity (Andersen et al., 2006). Input specificity refers to the observation that when a tetanus (or another induction protocol) is applied to one of two (or more) inputs converging on the same post-synaptic target then the potentiation is only observed at the tetanised input. The synaptic strength at the untetanised input(s) is unaltered. This shows that LTP is not some global change at the level of the neuron but a highly localised change at the level of the input. Commonly, this is assumed to equate to the tetanised synapses. Of course, a synaptic modification increases the storage capacity of a network of neurons enormously compared to a neuron wide change. So the input specificity of LTP is an attractive property, as well as an experimentally convenient feature. Co-operativity refers to the property that there is a threshold number of inputs that need to be activated simultaneously for LTP to occur. Associativity is an extension of both input specificity and co-operativity. It refers to the property whereby a weak tetanus (defined as one that is subthreshold for inducing LTP) will successfully induce LTP if paired with a strong tetanus applied to an independent input. This property is often equated with associative learning.

These features of LTP are remarkable and perfectly suited for a synaptic process that is involved in information storage. So a key question at the beginning of the 1980s was how LTP was triggered and could the mechanism explain the key features of input-specificity, co-operativity and associativity?

## 3 NMDARs and the induction of LTP

The discovery of specific NMDAR antagonists in the 1970s, primarily in the laboratories of Hugh McLennan (McLennan and Lodge, 1979) and Jeff Watkins (Davies et al., 1981), opened the way to investigate NMDARs in synaptic function. The first role to be found was in “polysynaptic” transmission in the spinal cord (Biscoe et al., 1977). We were interested to know what role NMDARs may play in the hippocampus and, more generally, did any particular glutamate receptor subtype have any specific part in synaptic transmission and synaptic plasticity in this structure. We found that a specific NMDAR antagonist, d-2-amino-5-phosphonopentanoate (d-AP5, also known as d-APV), had no discernible effect on the synaptic response, evoked by low frequency stimulation, but prevented the induction of LTP at the SCCP in rat hippocampal slices (Collingridge et al., 1983b) (Fig. 1A). This observation has been repeated numerous times and extended to many other pathways in the CNS. However, NMDARs do not trigger the induction of LTP at all pathways in the CNS, as first shown at the mossy fibre projection from granule cells to CA3 neurons in the hippocampus (Harris and Cotman, 1986). (Subsequent work has shown that kainate receptors may function as the induction trigger at these synapses (Bortolotto et al., 1999)).

Given the observation that NMDAR antagonists block the induction of LTP, it was natural to ask whether NMDAR activation is sufficient to induce LTP. To explore this question we applied NMDA either locally to the dendrites, by ionophoresis (Collingridge et al., 1983b) (Fig. 1B), or globally, by bath application (Collingridge et al., 1983a) (Fig. 1C). Using either method we were not able to reliably induce LTP. Instead we observed a variety of different phenomena. We initially observed a marked depression of synaptic transmission, which was associated with a reduction in the presynaptic fibre volley. This was commonly followed by a rebound potentiation (RP) that declined back to baseline values in tens of minutes. Rarely did we observe a sustained potentiation that could be classified as a form of LTP. When we increased the duration of the NMDA application, to a minute or more, we consistently observed a long-term depression (LTD) of the synaptic response. We assumed that the failure to induce LTP by agonist application was either because there was the need for an additional trigger, in addition to NMDAR activation, or because LTP required NMDARs to be activated in a manner that was hard to reproduce by exogenous application of an agonist. So what does NMDA application do? Recently we have revisited NMDA induced synaptic plasticity and present previously unpublished observations later in this article.

## 4 The mechanism of induction of NMDAR-LTP

So how do NMDARs trigger the induction of LTP? We were aware of the findings of Evans and Watkins and their co-workers, that Mg^2+^ is a potent NMDAR antagonist (Ault et al., 1980) and so explored the consequence of removing Mg^2+^ from the perfusing medium (from the usual 1–2 mM) on synaptic transmission at the SCCP. We found that this resulted in a large contribution of NMDARs to the synaptic response induced by low frequency stimulation (Coan and Collingridge, 1985). This implied that Mg^2+^ was limiting the synaptic activation of NMDARs during low frequency stimulation. Did this mean that the ability of Mg^2+^ to block NMDARs was altered during high frequency stimulation? If so, how? We were aware of the work of John MacDonald that showed that the NMDAR-conductance has an unusual voltage-dependence (MacDonald and Wojtowicz, 1980) but the penny only dropped with the discovery by Ascher and colleagues that the ability of Mg^2+^ to block NMDAR responses, induced by agonist application to cultured neurons, was highly voltage-dependent and involved a rapid block and unblock at the level of single NMDAR channels (Nowak et al., 1984). The obvious implication of this discovery, also observed around the same time by Mayer et al. (1984), was that the Mg^2+^ block was transiently relieved during high frequency stimulation due to the depolarisation that occurs during a tetanus. We tested this directly by delivering high frequency stimulation and recording the synaptic response and indeed were able to observe the predicted NMDAR mediated synaptic component (Herron et al., 1986). This was observed as a slow synaptic potential that summated with the fast, AMPAR-mediated EPSPs in a temporal manner.

However, the Mg^2+^ unblocking rate is fast and one would expect full unblocking during an AMPAR-EPSP, which raised the question as to why NMDARs did not contribute considerably to the low frequency synaptic response? An important clue was provided by the study of Dale and Roberts, who showed that NMDARs mediated a slow, monosynaptic response in the spinal cord (Dale and Roberts, 1985). We reasoned that the answer may lie in the kinetics of the NMDAR-synaptic response. We therefore compared the time course of the AMPAR and NMDAR mediated EPSCs in response to single shock stimulation, by holding the neuron at a membrane potential where the Mg^2+^ block was minimised. We observed that the NMDAR-EPSC was much slower than the AMPAR-EPSC to be activated (Collingridge et al., 1988). Thus the Mg^2+^ block is not removed from NMDAR channels during the depolarisation imparted by a single AMPAR-EPSC because of their slow gating. Our initial studies required the NMDAR-EPSC to be estimated by subtraction of responses in the presence and absence of d-AP5, since available antagonists did not permit selective block of AMPARs. But around that time, the quinoxalinedione antagonists, such as CNQX, were described by Honoré and colleagues (Honoré et al., 1988). We therefore tested the prediction that with AMPARs blocked, NMDARs could support a slow, monosynaptic response (Blake et al., 1988; 1989; Davies and Collingridge, 1989). A similar observation was made independently (Andreasen et al., 1989) and rapidly confirmed by other groups (Hestrin et al., 1990; Kessler et al., 1989; Konnerth et al., 1990; Sah et al., 1990). With AMPARs blocked pharmacologically, the voltage-dependence of the monosynaptic NMDAR-EPSC in the presence of Mg^2+^ is readily observed.

## 5 A key role for synaptic inhibition

During the induction of LTP there is another important factor that is relevant to the synaptic activation of NMDARs, namely synaptic inhibition. Thus although the time-course of an AMPAR-EPSC is relatively brief, the EPSP it generates decays much more slowly due to the time-constant of the neuron. An AMPAR-EPSP would therefore be expected to remove the Mg^2+^ block quite effectively and enable NMDARs to contribute to the synaptic response. However, when multiple fibres are activated synchronously the EPSP is curtailed by a GABA-mediated IPSP, which hyperpolarises the neuron to a region where the Mg^2+^ block of NMDARs is intensified and prevents a noticeable contribution of NMDARs to the synaptic response. This is simply demonstrated by pharmacological inhibition of GABA-A receptors, when NMDARs contribute a slow component to the EPSP evoked during low frequency transmission (Dingledine et al., 1986; Herron et al., 1985). (Note that synchronous activation of fibres is a natural firing pattern in the hippocampus that is needed to exceed the co-operativity threshold).

So why, during high frequency stimulation, does temporal summation of GABAR-mediated IPSPs not counteract the depolarisation provided by temporal summation of AMPARs EPSCs? During low frequency transmission GABA-A dominates over the EPSP to move the membrane potential towards ECl^−^. Linear addition of the AMPAR and GABA-AR responses would predict that the neuron would not obtain a membrane potential where the Mg^2+^ block of NMDARs is appreciably removed. However, there are other changes that occur, in addition to summation of synaptic potentials, during high frequency stimulation. First, GABAR-inhibition is labile. Following the release of GABA from a nerve terminal, some of the GABA activates inhibitory GABA-B autoreceptors to inhibit subsequent GABA release. This results in a profound inhibition of both the GABA-A and GABA-B components of the IPSP (Davies et al., 1990). Second, there is a depolarising shift in the reversal potential of the GABA-AR response. Third, there is a build up of extracellular K^+^, which depolarises the postsynaptic membrane. The relative contribution of these, and potentially other, factors is complex and varies according to the stimulus parameters employed (Davies and Collingridge, 1993).

These studies have demonstrated that the complex interplay between AMPAR and GABAR EPSPs determine how and when NMDARs contribute to the synaptic response and trigger the induction of LTP. But what happens under more physiological conditions? The use of a tetanus to induce LTP (Andersen et al., 1977) is a convenient experimental paradigm with which to explore its mechanisms but is unlikely to closely mimic the physiological situation. In the exploring animal, CA3 neurons tend to fire in brief high frequency synchronised discharges with an interval that is equivalent to the theta rhythm. When these parameters are applied to hippocampal slices (theta-burst stimulation, TBS), LTP is readily induced (Larson and Lynch, 1986; Larson et al., 1986). We have used one such protocol, priming (Diamond et al., 1988), to explore the roles of inhibition in regulating the synaptic activation of NMDARs. We found that a single priming stimulus followed 200 ms later by four shocks delivered at 100 Hz readily induced LTP and that the induction of this form of LTP was prevented by a GABA-B antagonist (Davies et al., 1991). As predicted, the induction of LTP correlated with the presence of an NMDAR-mediated component of the synaptic response. Thus, the role of GABA-B autoreceptors in regulating the synaptic activation of NMDARs, and hence LTP, is likely to be of physiological significance.

## 6 Visualising the Ca^2+^ signal associated with NMDARs and LTP

We speculated that the role of the NMDAR in LTP might have something to do with Ca^2+^, since early indications were that the NMDAR may be associated with a higher Ca^2+^ permeability than other glutamate receptors. Direct evidence for a key role of Ca^2+^ was the discovery that the chelation of Ca^2+^ in the postsynaptic neuron prevented the induction of LTP (Lynch et al., 1983). The subsequent discovery that NMDARs have a significant permeability to Ca^2+^ (MacDermott et al., 1986) led to the widely held assumption that LTP is triggered by the Ca^2+^ that permeates synaptically-activated NMDARs, which, assuming this process is restricted to activated synapses, can explain the property of input specificity.

**Fig. 2:**
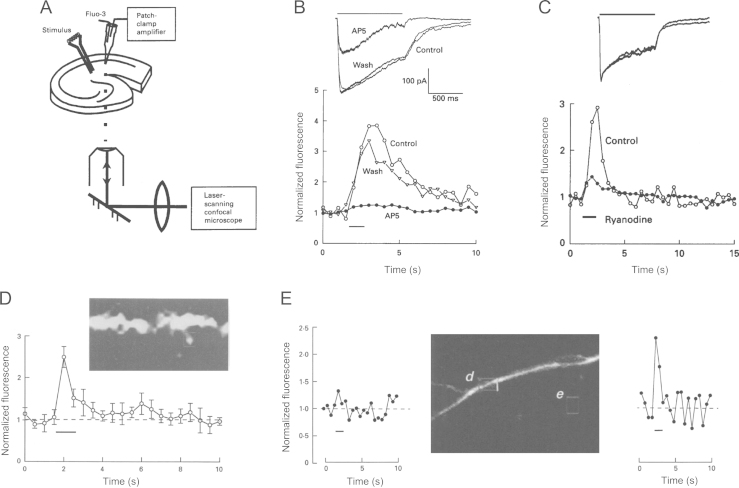
NMDAR-mediated Ca^2+^ entry during high frequency stimulation. (A) Schematic of the experimental arrangement. (B) AP5 reduces the synaptic current and eliminates the dendritic Ca^2+^ transient. (C) Ryanodine does not affect the synaptic current but substantially reduces the dendritic Ca^2+^ transient. (D) Ca^2+^ transients in individual dendritic spines. (E) Ca^2+^ transients are localised. All responses are from voltage-clamped CA1 neurons in response to HFS (100, Hz, 1 s). Modified from Alford et al. (1993).

To investigate these questions directly we tested whether it would be possible to image Ca^2+^ from neurons during their synaptic activation in hippocampal slices (Alford et al., 1993) (Fig. 2). By combining whole-cell recording with confocal microscopy we could detect Ca^2+^ entry into individual spines. Because using confocal microscopy we were close to the threshold for detecting Ca^2+^ signals in individual spines we generally integrated the signal over a small portion of dendrite, thereby averaging the signals from tens of spines. The synaptic Ca^2+^ transients that we observed were abolished by d-AP5 (Fig. 2B), but not CNQX, which is consistent with NMDARs acting as the trigger. Surprisingly, however, the Ca^2+^ transients were substantially reduced by treatments that depleted intracellular Ca^2+^ stores (Fig. 2C). This suggests that the Ca^2+^ that permeates NMDARs is magnified by Ca^2+^ release from intracellular stores.

The Ca^2+^ transients were rapid, particularly when measured from individual spines (Fig. 2D). Also, we observed marked heterogeneity in the response at varying spatial locations (Fig. 2E). These observations are consistent with the idea, though of course in themselves do not prove, that Ca^2+^ signalling restricted to individual postsynaptic elements underlies input specificity.

Since our initial studies, the imaging of synaptic Ca^2+^ transients at individual synapses has become routine and the imaging quality improved through the development of two-photon microscopy. Most significantly, the technique has been used to apply optical quantal analysis to address the locus of expression of LTP (Emptage et al., 2003) and to confirm the presence of pre-synaptic NMDARs at the SCCP (McGuinness et al., 2010).

## 7 The NMDAR confers synapses with unique properties

As has been discussed previously, the biophysical properties of the NMDAR provide an explanation for the hall-mark features of LTP; namely *input-specificity*, *co-operativity* and *associativity. Input specificity* is determined by the requirement for an active synapse to provide the necessary l-glutamate to activate NMDARs at the synapse. The release of l-glutamate at a single synapse is sufficient for LTP to be induced at that synapse, since when a neuron is artificially depolarised to remove the Mg^2+^ block then LTP can be induced even when only single fibres, and indeed single inputs, are activated. *Co-operativity* can be explained by the need to stimulate multiple inputs to provide sufficient natural depolarisation to remove the Mg^2+^ block as required to induce LTP. Thus, although the co-operativity requirement can be overcome experimentally by artificial depolarisation, synchronous activation of inputs is likely to be a major physiological means to achieve this. (The timing of single inputs with postsynaptic spiking is another). The need for synchronous activation may serve to prevent plastic changes in response to spurious inputs. *Associativity* is an extension of cooperativity, where an independent input helps a weak (i.e., subthreshold) input to induce LTP. In the present context, this could be anything that facilitates the synaptic activation of NMDARs. Conceptually, any input that favours depolarisation over hyperpolarisation could serve this function. The reduction in GABAR inhibition, conferred by GABA-B autoreceptors is one example. The inhibition of K^+^ conductances by, say, ACh would be another. Of course, associative mechanisms could also occur independently of the membrane potential, for example by affecting the NMDAR-conductance directly.

## 8 STP and LTP are distinct forms of NMDAR-mediated synaptic plasticity

**Fig. 3:**
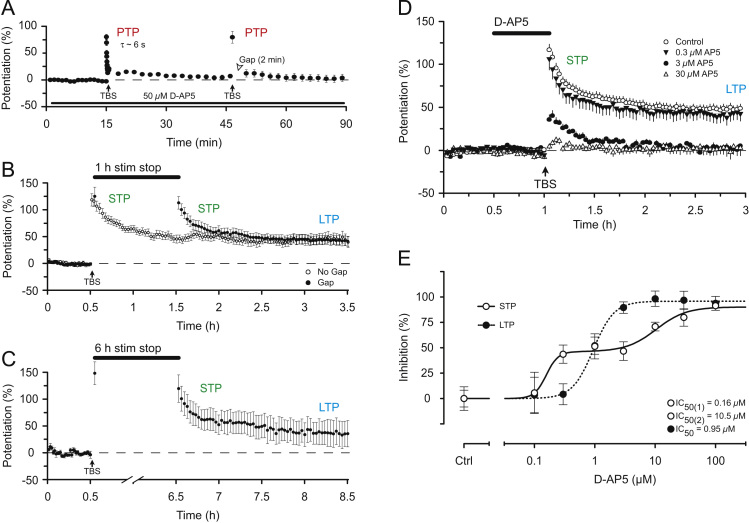
PTP, STP and LTP. (A) PTP is recorded in the presence of D-AP5 and decays passively (modified from Volianskis and Jensen (2003)). (B) STP decays in an activity-dependent manner. The time-course plots compare the response to TBS with no pause in stimulation or a 1 h pause in stimulation (modified from Volianskis and Jensen (2003)). (C) STP can be stored for at least 6 h (modified from Volianskis and Jensen (2003)). (D) D-AP5 differentially affects STP and LTP. (E) Concentration–response curves for D-AP5 antagonism define two components of STP and one of LTP. Modified from Volianskis et al. (2013a).

HFS and TBS evoke a synaptic potentiation that has several mechanistically distinct components (Bliss and Collingridge, 2013). Initially there is a brief, rapidly decaying component that is resistant to NMDAR blockade and is termed post-tetanic potentiation (PTP) (Figs. 1A, 3A). This is followed by a decaying phase of potentiation that is commonly referred to as short-term potentiation (STP), and a stable phase of potentiation that is usually referred to as LTP (Fig. 3B), both of which are sensitive to NMDAR blockade (Figs. 1A, 3A). In a recent article we defined three components of LTP: LTPa, LTPb and LTPc corresponding to STP, protein synthesis-independent LTP and protein synthesis-dependent LTP, respectively (Park et al., 2014). Here we refer to STP (LTPa) and LTP, where LTP corresponds to LTPb.

STP has a remarkable property, which distinguishes it from both PTP (Fig. 3A) and LTP (Fig. 3B), in that it decays in an activity-dependent manner (Volianskis and Jensen, 2003). This can be clearly observed when synaptic stimulation in stopped. The level of STP is stored until stimulation is resumed, at which point it continues to decay at the same rate (Fig. 3B). STP can be stored for remarkably long periods of time. Fig. 3C shows examples where STP was stored for 6 h. Furthermore, STP and LTP have different functional consequences for the transfer of synaptic information in that STP modulates the frequency response (Volianskis et al., 2013b) whereas LTP provides amplification whilst preserving the fidelity of synaptic transmission (Pananceau et al., 1998; Selig et al., 1999; Volianskis et al., 2013b).

STP and LTP can also be distinguished on the basis of their sensitivity to NMDAR antagonists. In the initial study with AP5 (Collingridge et al., 1983b), STP seemed less sensitive than LTP to antagonism (Fig. 1A), an effect that was substantiated when more quantitative experiments were subsequently performed (Malenka, 1991; Volianskis et al., 2013a) (Fig. 3D). In a more recent analysis of the sensitivity of STP and LTP to AP5 we uncovered an unexpected complexity. We found that whereas LTP was a unitary phenomenon, with respect to its sensitivity to AP5, STP comprised two distinct components of roughly equal magnitude (Volianskis et al., 2013a). One component of STP was more sensitive and the other component of STP was less sensitive than LTP to the effects of AP5. We defined these two components as STP(1) and STP(2), respectively. The IC_50_ values, calculated from full concentration response curves were as follows: STP(1)=0.16 µM, LTP=0.95 µM, STP(2)=10.5 µM (Fig. 3E). (Retrospectively, one can conclude that the residual STP observed in the presence of AP5 in Fig. 1A. is STP(2)).

STP(1) and STP(2) are not only pharmacologically distinct but they are also physiologically distinct processes too. Both require synaptic stimulation for their decay but STP(1) decays more quickly than STP(2) (Volianskis et al., 2013a). Accordingly, STP(1) contributes more to the peak and STP(2) more to the later decay of STP. Since both components of STP decay in an activity-dependent manner it is possible that, despite their differing sensitivities to d-AP5, there is some convergence in their mechanisms.

## 9 STP and LTP involve different NMDAR subtypes

**Fig. 4:**
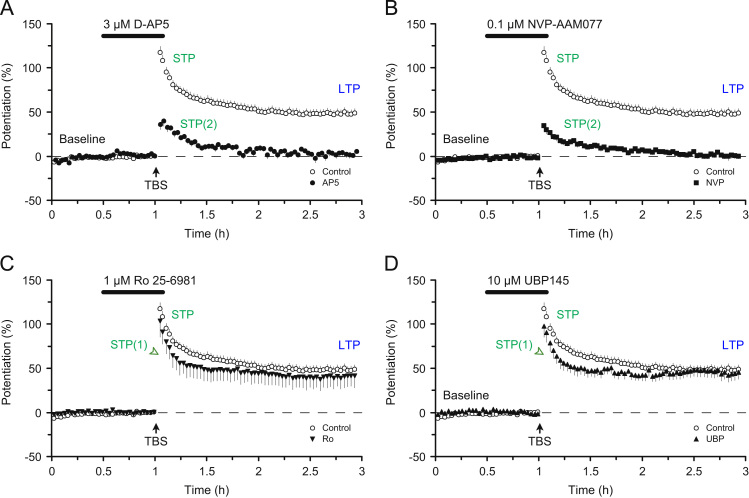
NMDAR subtype-dependence of STP and LTP. (A) AP5 selectively antagonises one component of STP (STP1) and LTP. (B) NVP resembles AP5. (C) Ro selectively antagonises one component of STP (STP2). (D) UBP resembles Ro. Synaptic plasticity was triggered using theta burst stimulation (TBS). Modified from Volianskis et al. (2013a).

**Fig. 5:**
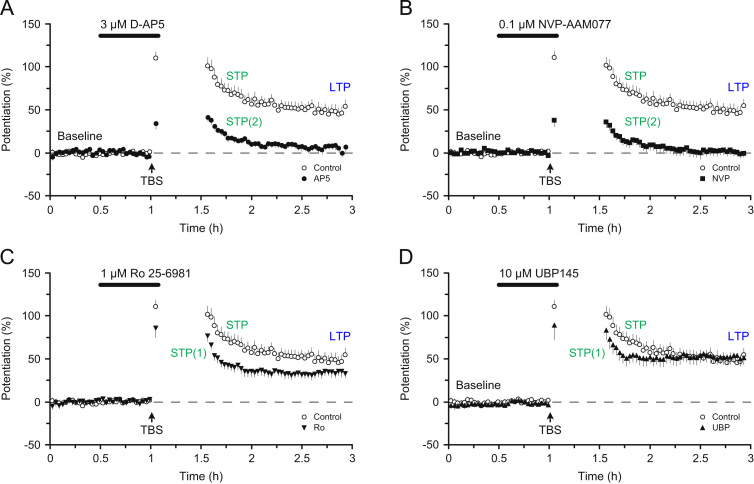
NMDAR subtype-dependence of STP and LTP. Experiments are identical to those presented in Fig. 3 except a 30 min pause in stimulation was introduced shortly after the delivery of TBS. Modified from Volianskis et al. (2013a).

More recently, with the introduction of more subtype-selective NMDAR antagonists, we have investigated the role of different NMDAR subtypes in the two components of STP and LTP in slices obtained from adult rats (Volianskis et al., 2013a). On the basis of the outcomes of quantitative pharmacological experiments using both expressed receptors of known subunit composition and native synaptic receptors we selected four antagonists, AP5, NVP, Ro and UBP, which have differing degrees of subtype specificity. Our basic finding is illustrated in Fig. 4. What this shows is that, at the concentrations employed, AP5 and NVP block STP(1) and LTP but not STP(2) whereas Ro and UBP selectively block STP(2). An identical result was obtained when the experiments were performed with a 30 min gap in stimulation (Fig. 5). Even without any knowledge of the pharmacology of these compounds one can conclude that STP(2) has a different NMDAR subtype dependence compared with STP(1) or LTP. By comparing the sensitivity of the three components of synaptic plasticity to the results from full concentration–response curves on native and cloned receptors for the four antagonists we could make the following inferences: LTP is mediated by a mixture of triheteromeric NMDARs comprised of GluN1, GluN2A and GluN2B subunits (N1/2A/2B) and diheteromeric NMDARs (N1/2A), with the former dominating. STP(1) has a similar, and perhaps identical, subunit dependence. In stark contrast, STP(2) is mediated by GluN2B and GluN2D subunits (potentially as a N1/2B/2D triheteromer). For a fuller description of the roles of different NMDAR subtypes in STP and LTP the reader is referred to Volianskis et al. (2013a).

## 10 NMDA-induced rebound potentiation (RP) is distinct from STP

**Fig. 6:**
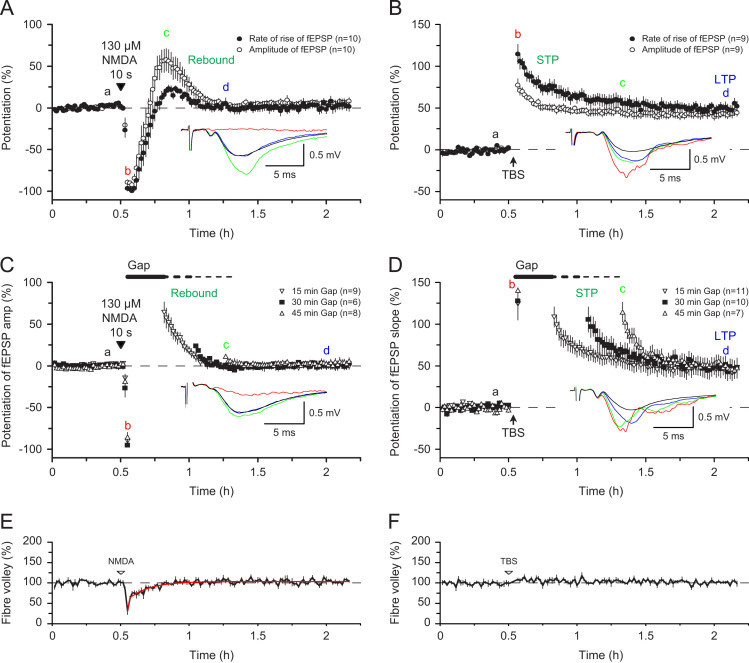
NMDA induced rebound potentiation (RP). (A) RP is associated with a larger effect on the peak compared to the slope of the fEPSP. (B) STP is associated with a larger effect on the slope compared with the peak of the fEPSP. (C) RP decays passively. (D) STP decays actively. E. NMDA depresses the FV. (F) TBS has no effect on the FV. (Previously unpublished).

In our early experiments (Collingridge et al., 1983b), we found that a brief application of NMDA was able to induce a rebound potentiation (RP) (Fig. 1B), which has a similar duration as STP (Fig. 2B). This led us to wonder whether the two processes may be mechanistically related. To investigate this issue we have now compared STP and RP in interleaved experiments and found that STP and RP are clearly distinct forms of plasticity (Fig. 6). First, RP is mainly observed as an increase in the amplitude of the fEPSP, with only a small effect on the slope (Asztely et al., 1991; McGuinness et al., 1991) (Fig. 6A). In contrast, STP is associated with a larger change in the slope than the amplitude (Fig. 6B). Second, RP cannot be stored during a pause in stimulation but decays passively (Fig. 6C). This is again in contrast to STP where the response is stored in the absence of synaptic activation (Fig. 6D). Although further work is required to understand the origins of RP, it can be concluded that RP does not evoke the same mechanisms as STP (or LTP).

NMDA-induced RP is preceded by a transient depression of synaptic transmission (Fig. 6A). The effect is associated with a depression of the presynaptic fibre volley (FV), suggesting that it is, at least in part, presynaptic in origin (Fig. 6E). In contrast, there is no change in the FV when TBS is applied (Fig. 6F). As discussed previously, the depression of the FV could be due to an action of NMDA on presynaptic NMDARs, due to an increase in extracellular K^+^ resulting from the postsynaptic activation of NMDARs or both (Collingridge, 1992; Collingridge et al., 1991; Park et al., 2014; Shih et al., 2013).

## 11 NMDA-induced LTD and synaptically-induced LTD

**Fig. 7:**
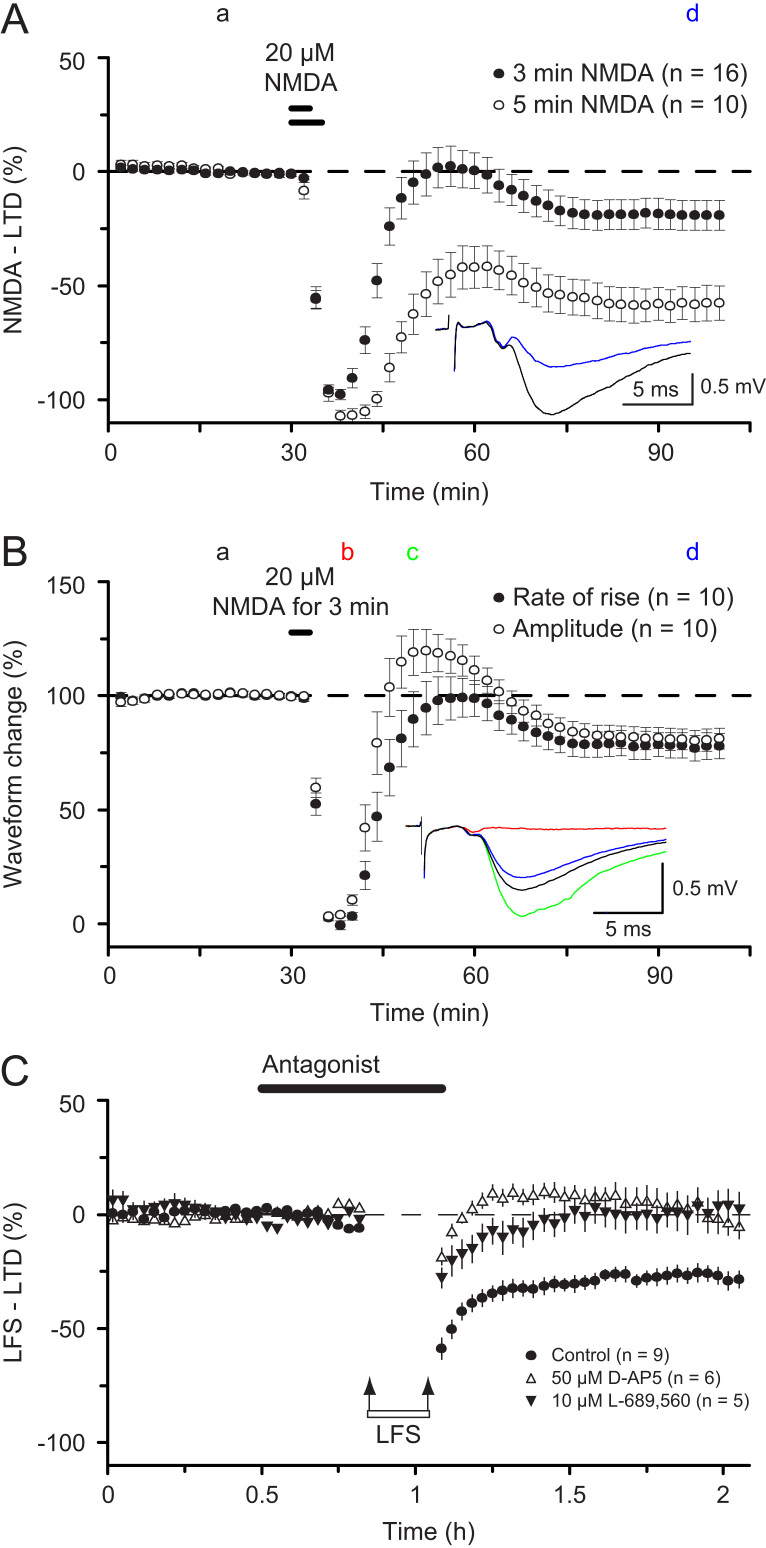
NMDAR-LTD. (A) NMDA-induced LTD increases with the duration of NMDA application and is not associated with changes in the FV after the recovery from transient depression. (B) NMDA-induced LTD is associated with equal changes in fEPSP slope and amplitude. (C). D-AP5 and L-689,560 both block the induction of LFS-LTD. LFS comprised 1 Hz, stimulation for 15 min. (Previously unpublished).

When the duration of the application of NMDA is increased then RP (still seen in Fig. 7A and B) gives way to a form of LTD (Collingridge et al., 1983a) (Fig. 7A). Unlike RP, NMDA-induced LTD involves similar changes in fEPSP amplitude and slope (Fig. 7B) and occurs independently of alterations in the FV, which is depressed initially (Fig. 7A). In these regards NMDA-LTD resembles NMDAR-LTD, as induced by low frequency stimulation (LFS, 900 shocks at 1 Hz, (Dudek and Bear, 1992)). It has been shown that NMDAR-LTD induced by synaptic stimulation and LTD induced by exogenous NMDA share similar mechanisms (Lee et al., 1998), though there may be mechanistic differences too.

Recently, it has been proposed that, unlike LTP, LTD induced synaptically does not require permeation of Ca^2+^ through NMDARs, but rather it involves a metabotropic action of NMDARs (Nabavi et al., 2014; 2013). This claim was based on the insensitivity of LTD to 7-chlorokynurenic acid (7-ClKA), a glycine-site antagonist of the NMDAR that we had previously shown to block the induction of LTP (Bashir et al., 1990). Given the implications of such a claim, we re-investigated whether inhibition of the glycine site affects induction of LTD. Rather than use 7-ClKA, which is a weak and non-specific glycine site antagonist, we used L-689,560 (Grimwood et al., 1995), which is a potent and highly specific glycine site antagonist. We found that L-689,560 consistently and completely prevented the induction of LTD (Fig. 6F). We assume that the difference is that during LFS there is glycine and/or d-serine release which out-competes 7-ClKA from the glycine co-agonist site. In conclusion, we do not consider that there are grounds to challenge the widely held view that Ca^2+^ permeation through NMDARs is the trigger for NMDAR-LTD.

## 12 Concluding remarks

So to return to the question of whether NMDAR=LTP=Memory, what can we conclude?

**Fig. 8:**
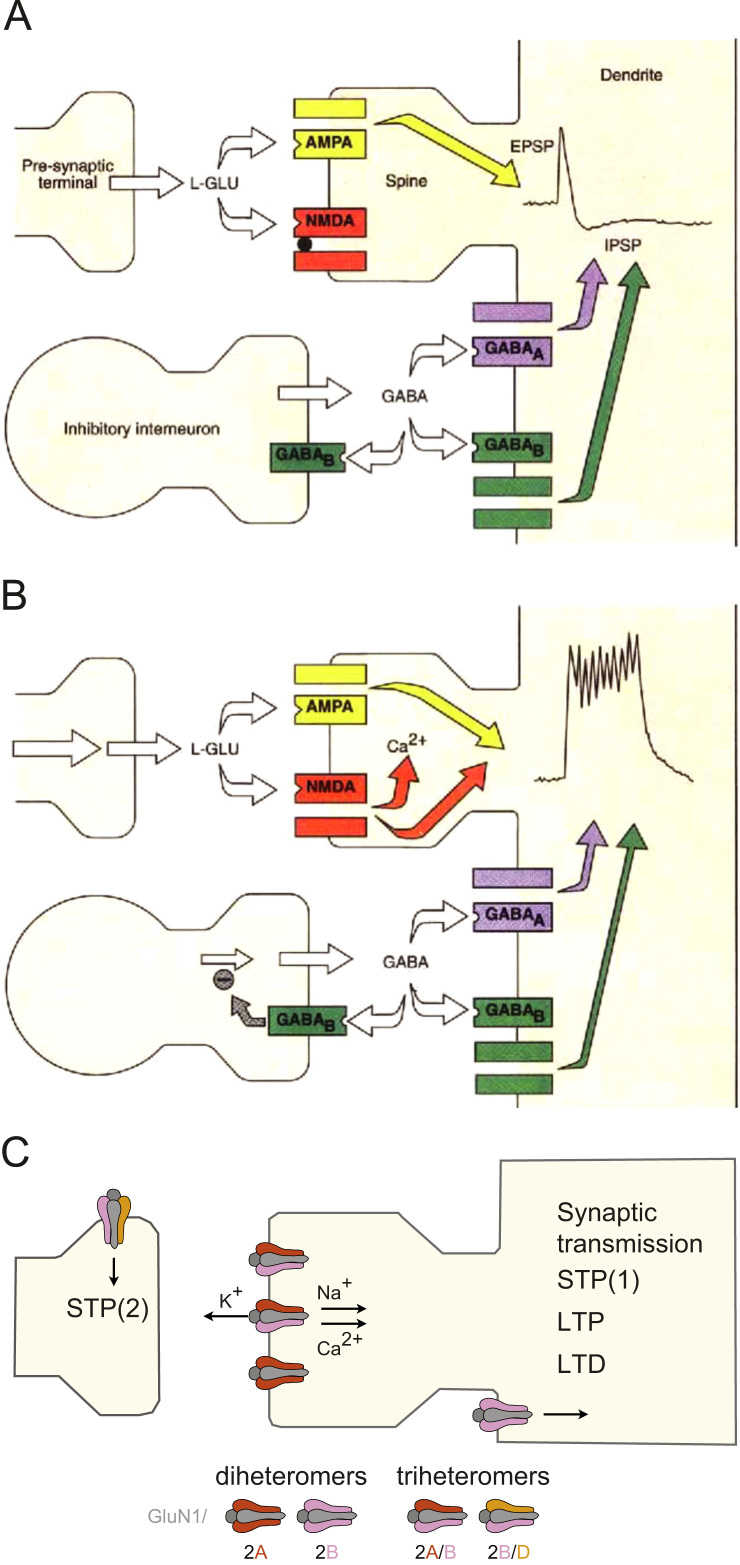
Various functions of NMDA receptors. (A) Release of glutamate during low frequency synaptic transmission leads to activation of AMPARs (EPSP) and sparse activation of NMDARs, which is insufficient to induce synaptic plasticity. The predominantly AMPAR-mediated EPSPs are shaped by GABAergic interneurons through GABA acting on GABA_A_ and GABA_B_ receptors (IPSP) that prevent over-activation of NMDARs (Bliss and Collingridge, 1993). (B) Release of glutamate during high-frequency synaptic transmission leads to activation of NMDARs due to relief of Mg^2+^ block. This happens because of summation of AMPAR-EPSPs, depolarisation that is mediated by build-up of extracellular K^+^ and GABA_B_ auto-receptor mediated inhibition of GABA release (Bliss and Collingridge, 1993). (C) Activation of NMDARs triggers a variety of different forms of synaptic plasticity. STP is expressed presynaptically, as an increase in P(r). It comprises two components: STP(2) is induced via activation of GluN2B and 2D containing NMDARs (potentially as a triheteromer located on the presynaptic terminal). STP(1) is induced via activation of GluN2A/B receptors. These could be located postsynaptically and signal (together with AMPARs) to the presynaptic terminal via their flux of K^+^ (Collingridge, 1992; Park et al., 2014; Shih et al., 2013). LTP is induced via activation of GluN2A and GluN2B receptors, with a triheteromer being the dominant species. LTD may involve different subtypes of NMDAR, including diheteromeric GluN2B receptors (Liu et al., 2004).

The simple answer is No. This is clearly illustrated by some established facts. First, the synaptic activation of NMDARs does not invariably induce LTP (Fig. 8). NMDARs are important for synaptic transmission. Their contribution is readily apparent during high frequency discharges, even very brief ones that comprise just a few stimuli. At some synapses, or in some situations, this activation may not induce LTP. Even when it does, NMDARs are doing more than just inducing synaptic plasticity, they are contributing to the transmission at the synapse. Second, NMDARs induce other forms of synaptic plasticity, most notably STP, depotentiation and *de novo* LTD. These are also believed to contribute to memory. Third, LTP can be induced without the need to activate NMDARs as exemplified at the mossy fibre synapse. Even where NMDARs may mediate the induction of LTP ordinarily, such as at the SCCP, their activation can be by-passed, at least under certain experimental conditions, and LTP can be triggered by the activation of voltage-gated ion channels (Grover and Teyler, 1990), mGluRs (Bortolotto and Collingridge, 1993) and calcium permeable AMPARs (Jia et al., 1996).

So what is the physiological role of NMDARs? It would seem that it is multifaceted. One function is to contribute to high frequency transmission. A second is to trigger the induction of some, but not all, forms of synaptic plasticity. Often these two may be intricately linked and in that sense one can conclude that NMDAR~LTP.

As has often been said before, the NMDAR can act as a co-incidence detector requiring both presynaptic activity, to release l-glutamate, and postsynaptic activity, to provide the depolarisation required to alleviate the Mg^2+^ block of NMDARs, which endows synapses with “Hebbian-like properties”. So it is perhaps better to view the NMDAR as a co-incidence detector, one key function of which is in the induction of what is probably the major form of synaptic plasticity in the CNS.

We can conclude that:

• NMDARs are important for many forms of LTP.
• NMDARs do more than just trigger the induction of LTP.
• LTP is important for learning and memory.
• LTP does more than just contribute to learning and memory (in the conventional sense, at least).
• There is more to learning and memory than LTP.
• There is more to learning and memory than NMDARs.

But we end by stating that the NMDAR–LTP–Learning and Memory association is a powerful one that has served neuroscience well and will continue to do so. We can therefore perhaps conclude with:

NMDA~LTP~Memory

## 13 Experimental procedures

Animal experiments were performed after institutional approval, according to the UK Scientific Procedures Act, 1986 and European Union guidelines for animal care. Animals were killed by cervical dislocation after isoflurane anaesthesia and death was confirmed by a permanent cessation of the circulation (Schedule 1). STP, RP and NMDA induced LTD experiments were performed on hippocampal slices from adult Wistar rats as described previously in Volianskis et al. (2013a), Volianskis and Jensen (2003). Low frequency stimulation induced LTD experiments were performed on P14 slices from Wistar rats as described previously in Bartlett et al. (2007). Chemicals were from Ascent (Bristol UK), Tocris (Bristol UK), Fisher Scientific (Loughborough, UK) or Sigma (Dorset, UK).

## 14 Conflict of interest statement

The authors declare that they have no competing interests.

## Author contribution

AV – performed and analysed experiments, wrote the manuscript.
GF – performed and analysed experiments.
MSJ – supervised and participated in research.
ZAB – supervised and participated in research.
DEJ – supervised and participated in research.
GLC – supervised, participated in research and wrote the manuscript.
